# Dolly at 25… is she ‘… still goin’ strong?’

**DOI:** 10.1530/REP-21-0212

**Published:** 2021-05-25

**Authors:** Kevin D Sinclair

**Affiliations:** 1 School of Biosciences, University of Nottingham, Loughborough, Leicestershire, UK

The birth of ‘Dolly the sheep’ (the first animal to be cloned from an adult (somatic) cell) on 5^th^ July 1996 marked a seminal moment in the field of developmental genetics for several reasons. It first provided unequivocal evidence of genomic equivalence between embryonic and somatic cells by demonstrating that it is possible to re-establish a pluripotent state in differentiated cells. In so doing, it inspired others to later identify at least some of the factors required for the induction of pluripotency in cultured somatic cells leading to the creation of ‘induced’ pluripotent stem cells (iPSCs) for therapeutic applications in both human and animal medicine (Takahashi & Yamanaka 2006). However, it was the need to develop an effective method by which to produce genetically modified (GM) livestock that drove the programme of work that ultimately led to the birth of Dolly. Existing GM approaches at that time relied on techniques, such as pronuclear DNA microinjection. These techniques were both inefficient and limited in their ability to introduce genetic change in large animals. Furthermore, in contrast to mice (Evans & Kaufman 1981, Martin 1981), the derivation and establishment of germ-line competent embryonic-stem cells (so amenable for introducing genetic modification in that species) has thus far remained elusive in livestock species. Thus, the approach adopted by Wilmut *et al.* (1997) involved the transfer of nuclei from somatic (i.e. cultured mammary) cells to enucleated oocytes followed by electrofusion and activation; a procedure referred to as somatic-cell nuclear transfer (SCNT).

The success of Dolly led quickly to a succession of papers reporting the birth of GM livestock derived from genetically altered somatic cells (e.g. Schnieke *et al.* 1997, Cibelli *et al.* 1998, Dai *et al.* 2002). However, both ethical and biological concerns regarding the underlying cloning procedure were raised at the time. Biological concerns related to the overall efficiency of SCNT, *in utero* and perinatal losses, together with the ‘biological’ age, longevity and health of cloned offspring; all of which threatened the prospect of generating GM animals by this means. *In utero* losses, developmental abnormalities and neonatal morbidity were attributable, at least in part, to the culture of gametes and embryos, which contribute independently to epigenetic dysregulation at both imprinted and non-imprinted loci (Young *et al.* 2001, Chen *et al.* 2017), leading to a phenomenon known as the large offspring syndrome (Young *et al.* 1998). Concerns were raised specifically about the health of Dolly, as she was diagnosed with osteoarthritis (OA) of the left stifle at a relatively young (5½ years) age (Rhind *et al.* 2004). It was suggested that she might have aged prematurely, and terminal fragment restriction analyses of her genomic DNA appeared to support the concept of telomere shortening (Shiels *et al.* 1999). However, this observation was at odds with those from other SCNT studies which generally found telomeres to ‘rejuvenate’ during nuclear reprogramming (Marión & Blasco 2010). Furthermore, our own retrospective radiographic assessments of the skeletons of Dolly, Megan and Morag (the latter two sheep had been cloned previously from differentiated cells; Campbell *et al.* 1996) reported a prevalence and severity of OA no different to that of naturally conceived sheep of comparable age (Corr *et al.* 2017). Indeed, several studies over the years have concluded that cloned offspring which survive beyond the neonatal period are healthy, age normally, produce viable offspring and animal products safe for human consumption (Lanza *et al.* 2001, Yang *et al.* 2007, Watanabe 2013, Sinclair *et al.* 2016). Yet concerns relating to animal welfare remain, and these have been sufficient to enforce a ban on commercial farm-animal cloning within the UK and EU, although not within the US and many other countries.

The consequence of this ban is that, in an era of comparatively well-funded iPSC research, the ability to undertake studies to improve the overall efficiency, safety and application of SCNT has been impeded. Yet, despite the global investment in iPSC research over the last 20 years, its potential for cell-based therapy has yet to be realised (Yamanaka 2020). Other commentators emphasise the merits of zygotic genome editing, using designer nucleases such as CRISPR/Cas9, to generate GM livestock, thus obviating the need for the more technically demanding SCNT (Tan *et al.* 2016). However, embryo mosaicism and low and variable germ-line transmission present challenges with this approach, particularly when multiplexed gene editing is required (Tanihara *et al.* 2021). Therefore, 25 years on, this anniversary issue of *Reproduction* addresses the question: is there still a role for SCNT in reproductive and regenerative medicine? It also considers how our understanding of the molecular mechanisms that underlie pregnancy failure and neonatal loss has improved over the past 25 years, describing refinements to procedures that impact on the overall efficiency of SCNT, whilst discussing residual ethical and societal concerns related to use of advanced reproductive technologies in animals and humans.

The issue (Video 1) opens with a brief historical perspective of the scientific legacy of Dolly from one of the co-authors (Prof Dr Angelika Schnieke) of the article that first reported this development in 1997. Klinger & Schnieke (2021) summarise current applications of SCNT and discuss its relevance in the 21st century. There then follows two articles that each provide both a historical and contemporary perspective on the role of SCNT in the generation of GM livestock. The first article (Polejaeva 2021) provides a detailed overview of the technical advances made in GM technologies over the past 25 years from a leading member of the team that produced the first cloned pigs by SCNT in 2000. This article also offers a perspective on the current and future role of SCNT in the creation of genetically engineered animals; a perspective that is extended by Galli & Lazzari (2021) who consider applications in several large-animal species together with intellectual-property and regulatory challenges that will ultimately determine its use.

Video 1 Professor Kevin Sinclair provides a video introduction to this anniversary edition on cloning by somatic-cell nuclear transfer. This video (https://doi.org/10.1530/REP-21-0212.
Download Video 1

The anniversary issue next considers the science underpinning SCNT. The establishment of a pluripotent state following nuclear transfer requires significant remodelling of inherited chromatin to occur within a matter of hours following reconstruction. These aspects are discussed for both farm and companion animals (Loi *et al.* 2021), and mice (Ogura *et al.* 2021), with emphasis placed on epigenetic modifications to chromatin required to induce a pluripotent state. Consideration is also given to the molecular events involved in chromatin remodelling in male and female gametes in the lead-up to syngamy during natural conception. It is based on the premise that a better understanding of these processes may lead to innovative approaches that will improve the overall efficiency of nuclear reprogramming.

The penultimate article in this series (Alberio & Wolf 2021) returns to the topic of GM livestock, considering further applications in animal breeding and the development of large-animal models for early human development and disease. The prospect of generating embryonic stem cells in farm animals may obviate the need for somatic cells and improve the overall efficiency of GM-livestock production, superseding current zygotic genome-editing approaches. The transfer of nuclei from such cells, rather than the creation of chimeric embryos, could then emerge to become the most effective means by which to establish GM-founder stock.

Finally, reference was made earlier in this editorial to ethical concerns raised following the birth of Dolly in 1996. The concluding article in this anniversary edition considers these issues and proposes that many of the concerns raised at that time persist to this day, although the context has somewhat shifted towards the use of genetic technologies that influence inheritance in both humans and farm animals (Greenfield 2021). The point is made that scientific and political judgements of what might be in the public interest may not necessarily be what the public wants or would choose. At the very least, it would seem that some 25 years later, the debate around the safe and ethical use of advanced reproductive technologies in human medicine and livestock production is ‘…still goin’ strong’.

## Declaration of interest

The author declares that there is no conflict of interest that could be perceived as prejudicing the impartiality of this editorial.

## Funding

This research did not receive any specific grant from any funding agency in the public, commercial or not-for-profit sector.
